# Supplementary material for: Impacts of Land-Use Change on Ecosystem Services Value in the South-to-North Water Diversion Project, China
Source: Int J Environ Res Public Health. 2023 Mar 13;20(6):5069. doi: 10.3390/ijerph20065069 (PMC10049115; doi:10.3390/ijerph20065069)
Supplement: Supplementary file 1 [file ijerph-20-05069-s001.zip › ijerph-2215730-supplementary.pdf]

Table S1 Provinces included in four regions.

Region	HAER	RAER	HAMR	RAMR
The included provinces	Shanghai	Shandong	northwestern Hubei	Beijing
	Zhejiang	eastern Hebei	southeastern Shaanxi	western Tianjin
	southern Jiangsu	eastern Tianjin	Southeastern Chongqing	northern Henan
		northern Jiangsu	southern Henan	western Hebei

* HAER-the headwater areas of the east route; RAER-the receiving areas of the east route; HAMR-the headwater areas of the middle route; RAMR- the receiving areas of the middle route.

Table S2 Areas of land use types in the study area from 2000 to 2020.

Subarea	Land use types	Index	2000	2005	2010	2015	2020
HAMR	Cultivated land	Area (km²)	41,818.23	41,398.50	41,265.69	40,913.52	40,483.62
		Area proportion (%)	30.16	29.78	29.68	29.47	29.17
	Forestry areas	Area (km²)	61,982.99	62,198.25	62,182.69	61,781.06	63,068.15
		Area proportion (%)	44.70	44.74	44.73	44.50	45.45
	Grassland	Area (km²)	29,999.92	30,178.46	30,176.83	30,153.68	28,876.97
		Area proportion (%)	21.63	21.71	21.71	21.72	20.81
	Water areas	Area (km²)	2350.48	2709.26	2742.24	2825.25	3079.02
		Area proportion (%)	1.69	1.95	1.97	2.04	2.22
	Built-up areas	Area (km²)	1786.86	1915.62	2005.02	2473.35	2678.47
		Area proportion (%)	1.29	1.38	1.44	1.78	1.93
	Unused land	Area (km²)	38.74	38.97	38.97	44.70	43.86
		Area proportion (%)	0.03	0.03	0.03	0.03	0.03
	Wetland	Area (km²)	699.88	584.20	610.72	640.01	536.18
		Area proportion (%)	0.50	0.42	0.44	0.46	0.39

HAER	Cultivated land	Area (km²)	34,340.51	32,754.78	31,390.14	30,347.18	27,397.84
		Area proportion (%)	65.16	61.90	59.32	57.51	51.97
	Forestry areas	Area (km²)	4157.01	4180.85	4175.38	4072.63	4080.03
		Area proportion (%)	7.89	7.90	7.89	7.72	7.74
	Grassland	Area (km²)	245.48	243.63	241.27	312.93	321.41
		Area proportion (%)	0.47	0.46	0.46	0.59	0.61
	Water areas	Area (km²)	7818.62	8079.11	8077.45	7947.56	7897.32
		Area proportion (%)	14.84	15.27	15.27	15.06	14.98
	Built-up areas	Area (km²)	5540.55	7153.34	8515.51	9543.12	12,436.03
		Area proportion (%)	10.51	13.52	16.09	18.09	23.59
	Unused land	Area (km²)	14.56	15.93	15.29	12.73	48.79
		Area proportion (%)	0.03	0.03	0.03	0.02	0.09
	Wetland	Area (km²)	582.17	491.60	497.84	531.39	541.87
		Area proportion (%)	1.10	0.93	0.94	1.01	1.03
RAMR	Cultivated land	Area (km²)	124,980.66	123,834.48	123,264.04	122,371.71	114,758.09
		Area proportion (%)	68.15	67.34	67.03	66.59	62.66
	Forestry areas	Area (km²)	21,474.06	21,583.05	21,587.86	21,415.50	21,745.74
		Area proportion (%)	11.71	11.74	11.74	11.65	11.87
	Grassland	Area (km²)	16,113.06	16,094.82	16,032.69	15,983.74	15,579.51
		Area proportion (%)	8.79	8.75	8.72	8.70	8.51
	Water areas	Area (km²)	2250.60	2396.37	2357.28	2423.49	3152.66
		Area proportion (%)	1.23	1.30	1.28	1.32	1.72
	Built-up areas	Area (km²)	16,849.76	18,308.47	18,927.21	19,818.09	26,705.43
		Area proportion (%)	9.19	9.96	10.29	10.78	14.58

	Unused land	Area (km²)	63.61	46.98	47.58	48.39	25.59
		Area proportion (%)	0.03	0.03	0.03	0.03	0.01
	Wetland	Area (km²)	1671.88	1640.75	1686.81	1701.15	1163.51
		Area proportion (%)	0.91	0.89	0.92	0.93	0.64
	Cultivated land	Area (km²)	127,693.87	126,531.32	125,176.01	124,206.44	121,978.53
		Area proportion (%)	70.50	69.53	69.03	68.50	67.33
	Forestry areas	Area (km²)	8320.24	8341.73	8343.06	8336.01	7538.46
		Area proportion (%)	4.59	4.58	4.60	4.60	4.16
	Grassland	Area (km²)	10,456.94	10,456.94	9793.74	9807.12	6772.29
		Area proportion (%)	5.77	5.75	5.40	5.41	3.74
	Water areas	Area (km²)	7597.55	8196.02	8223.47	8202.48	12,064.39
		Area proportion (%)	4.19	4.50	4.53	4.52	6.66
	Built-up areas	Area (km²)	22,422.98	24,536.37	26,004.42	26,963.16	30,181.36
		Area proportion (%)	12.38	13.48	14.34	14.87	16.66
	Unused land	Area (km²)	1941.77	1529.46	1398.19	1404.38	259.34
		Area proportion (%)	1.07	0.84	0.77	0.77	0.14
	Wetland	Area (km²)	2695.74	2376.99	2407.70	2406.11	2363.82
RAER		Area proportion (%)	1.49	1.31	1.33	1.33	1.30

Table S3 Land-use transfer matrix in the HAMR (km²).

Time	Type	Grass-land	Cultivated land	Built-up areas	Forestry land	Wet-land	Water areas	Unused land	Transfer out
2000–2005	Grassland	29,989.48	20.38	3.55	44.53	0	5.94	0	74.40

	Cultivated land	150.04	41,304.37	118.70	81.06	23.91	233.54	0	607.24
	Built-up areas	0.03	12.34	1772.45	0.03	0.97	4.62	0	17.99
	Forestry land	37.29	26.79	9.89	62,064.51	0	20.57	0	94.54
	Wetland	0.25	8.74	4.79	5.81	539.46	143.50	0	163.09
	Water areas	1.37	25.88	6.25	2.31	19.86	2301.09	0	55.68
	Unused land	0	0	0	0	0	0	38.97	0
	Transfer into	188.98	94.13	143.17	133.74	44.73	408.17	0	——
2005–2010	Grassland	30,163.58	3.10	0.48	9.26	0	2.03	0	14.87
	Cultivated land	6.15	41,215.94	77.31	27.78	7.23	63.12	0	181.59
	Built-up areas	0	12.16	1902.57	0.28	0.24	0.37	0	13.05
	Forestry land	7.09	17.61	20.80	62,144.72	2.53	5.37	0	53.41
	Wetland	0	1.83	0.44	0	568.20	13.73	0	16.00
	Water areas	0	15.05	3.42	0.66	32.52	2657.61	0.00	51.64
	Unused land	0	0	0	0	0	0	38.97	0
	Transfer into	13.25	49.75	102.44	37.98	42.52	84.62	0	——
2010–2015	Grassland	27,872.21	727.11	36.53	1449.98	19.17	36.10	2.25	2271.14

	Cultivated land	655.69	33,537.41	1341.33	4393.10	215.40	1050.78	11.95	7668.25
	Built-up areas	18.45	1023.28	703.54	91.98	26.88	137.87	0.07	1298.53
	Forestry land	1552.34	4485.12	200.31	55,482.04	64.93	304.55	5.89	6613.14
	Wetland	13.88	205.95	43.28	73.91	181.41	90.39	0	427.41
	Water areas	40.70	929.70	148.36	279.61	132.22	1204.55	2.61	1533.19
	Unused land	0.41	4.96	0	10.44	0	1.00	21.93	16.81
	Transfer into	2281.47	7376.11	1769.81	6299.02	458.60	1620.70	22.77	——
2015–2020	Grassland	14,107.03	6640.67	153.50	9036.17	15.32	142.08	5.59	15,993.32
	Cultivated land	6332.78	23,561.26	1599.00	7895.43	220.61	1264.19	6.01	17,318.02
	Built-up areas	115.74	1434.32	510.89	214.90	42.85	153.99	0	1961.80
	Forestry land	8158.69	7407.85	211.35	45,445.13	54.66	358.62	10.76	16,201.93
	Wetland	25.09	256.73	38.11	75.73	90.99	152.30	0.00	547.97
	Water areas	92.11	1133.88	157.01	325.54	111.30	1002.30	1.31	1821.16
	Unused land	5.55	9.24	0.03	9.22	0	0.47	20.19	24.51
	Transfer into	14,729.98	16,882.70	2159.00	17,556.98	444.73	2071.64	23.67	——

Table S4 Land-use transfer matrix in the HAER (km²).

Time	Type	Grass-land	Cultivated land	Built-up areas	Forestry land	Wet-land	Water areas	Unused land	Transfer out
2000–2005	Grass-land	231.50	7.13	4.32	2.00	0.35	1.83	0.68	16.31
	Culti-vated land	0.83	32,355.18	1684.70	40.97	3.75	317.98	0	2048.23
	Built-up areas	0.03	167.14	5371.07	0.50	0	14.25	0	181.92
	Forestry land	0	23.72	19.64	4129.19	0	0.73	0.68	44.78
	Wetland	1.42	35.86	22.76	0	474.82	69.62	0	129.66
	Water areas	9.21	121.74	49.28	0.08	7.08	7661.61	0	187.38
	Unused land	0	0	0	0	0	0	14.56	0
	Transfer into	11.48	355.59	1780.70	43.55	11.18	404.40	1.37	——
2005–2010	Grass-land	238.06	1.00	1.39	0.03	0	2.49	0	4.92
	Culti-vated land	0.64	31,129.10	1457.03	29.98	2.89	88.08	0	1578.62
	Built-up areas	0.03	171.65	6973.36	0.96	0	5.77	0	178.41
	Forestry	1.39	12.81	22.79	4135.30	0	0.38	0	37.3

	land								6
	Wetland	0	2.37	3.94	0.00	470.59	8.84	0	15.16
	Water areas	0.49	31.37	55.42	0.00	17.62	7960.30	0.39	105.29
	Unused land	0	0	0	1.00	0	0.03	14.90	1.03
	Transfer into	2.55	219.20	1540.59	31.97	20.51	105.59	0.39	——
2010–2015	Grass-land	168.58	8.02	8.99	45.57	0	4.84	0	67.41
	Culti-vated land	21.46	28,177.38	2204.53	454.30	43.15	390.34	0.40	3114.17
	Built-up areas	4.71	1228.92	7078.61	68.36	6.19	102.31	1.73	1412.21
	Forestry land	49.56	505.44	93.53	3463.63	2.56	39.59	1.87	692.55
	Wetland	8.73	21.74	6.41	4.28	393.50	46.58	0	87.74
	Water areas	59.85	368.28	147.86	28.84	84.90	7355.54	0.03	689.76
	Unused land	0	1.00	2.00	1.35	0	2.23	8.71	6.58
	Transfer into	144.30	2133.40	2463.32	602.70	136.80	585.88	4.02	——
2015–2020	Grass-land	57.10	68.78	46.02	47.82	27.52	64.55	0	254.68
	Culti-	83.65	21,647.36	5902.81	892.79	135.71	1570.29	28.09	8613

vated land								.33
Built-up areas	31.32	3290.35	5497.85	193.12	21.13	497.49	2.81	4036.21
Forestry land	46.93	787.80	240.59	2836.29	11.94	114.25	11.97	1213.47
Wetland	11.93	170.08	36.36	4.31	149.65	153.85	0	376.53
Water areas	85.59	1371.54	679.06	89.88	194.56	5481.88	5.28	2425.91
Unused land	0	4.32	1.62	4.63	0	1.51	0.64	12.09
Transfer into	259.41	5692.86	6906.46	1232.55	390.86	2401.94	48.15	——

Table S5 Land-use transfer matrix in the RAMR (km²).

Time	Type	Grassland	Cultivated land	Built-up areas	Forestry land	Wetland	Water areas	Unused land	Transfer out
2000–2005	Grassland	15,969.81	65.46	46.03	36.58	1.85	28.16	0	178.08
	Cultivated land	67.67	122,555.85	1985.46	108.11	75.12	420.72	3.09	2660.17
	Built-up areas	10.82	670.53	16,147.06	10.99	3.13	38.65	0.01	734.12
	Forestry land	23.99	85.80	57.37	21,338.12	4.30	15.02	0	186.48
	Wetland	2.77	113.57	7.93	11.89	1446.36	100.64	0	236.80
	Water areas	13.88	277.88	64.30	6.77	109.99	1790.71	0	472.82
	Unused land	0	17.28	0.20	0.16	0	2.33	43.88	19.98

	Transfer into	119.13	1230.52	2161.29	174.51	194.39	605.52	3.09	——
2005–2010	Grassland	15,990.72	15.07	41.15	32.79	6.59	2.61	0	98.22
	Cultivated land	20.40	122,970.89	663.51	19.71	38.63	72.29	0.72	815.24
	Built-up areas	0.25	127.86	18,176.10	0.37	0.31	3.47	0	132.25
	Forestry land	13.82	5.47	27.57	21,462.81	2.96	0	0	49.81
	Wetland	0.59	68.47	9.89	1.75	1518.49	41.10	0	121.80
	Water areas	1.04	28.49	8.88	0	119.83	2237.68	0	158.25
	Unused land	0	0.11	0	0	0	0	46.87	0.11
	Transfer into	36.10	245.48	751.00	54.62	168.32	119.46	0.72	——
2010–2015	Grassland	13,973.11	1256.88	134.59	584.08	32.71	32.77	5.39	2046.42
	Cultivated land	1109.91	11,3491.89	6510.02	1112.40	334.43	576.53	10.00	9653.29
	Built-up areas	85.51	5688.23	12,945.53	78.77	33.34	82.04	1.00	5968.90
	Forestry land	733.43	1115.37	88.14	19,500.20	45.01	19.62	1.64	2003.21
	Wetland	38.16	299.98	37.98	54.85	1205.38	48.34	0	479.31
	Water areas	38.30	479.42	100.83	19.16	50.22	1663.54	0	687.93
	Unused land	1.00	12.21	1.01	2.10	0	0.75	30.36	17.07
	Transfer into	2006.31	8852.09	6872.56	1851.36	495.72	760.06	18.03	——
2015–2020	Grassland	8364.39	3279.84	760.49	3112.80	140.34	182.13	7.85	7483.46

Cultivated land	3924.71	95,817.04	17,554.45	2709.78	535.87	1655.87	8.28	26,388.97
Built-up areas	437.11	11,356.95	7304.52	365.16	68.97	264.54	3.41	12,496.15
Forestry land	2545.86	2291.83	591.42	15,352.28	56.10	162.98	2.95	5651.13
Wetland	146.69	816.90	173.75	100.60	242.00	220.05	0.49	1458.49
Water areas	147.96	1098.10	296.74	91.80	119.08	665.56	1.02	1754.69
Unused land	7.62	31.09	3.76	3.09	1.00	0.24	1.59	46.79
Transfer into	7209.94	18,874.70	19,380.63	6383.23	921.36	2485.82	24.00	——

Table S6 Land-use transfer matrix in the RAER (km²).

Time	Type	Grass-land	Cultivated land	Built-up areas	Forestry land	Wet-land	Water areas	Unused land	Trans-fer out
2000–2005	Grassland	4098.49	3782.34	668.18	1389.92	130.53	267.08	120.75	6358.81
	Cultivated land	3373.82	109,831.12	9728.36	2213.42	452.69	1593.39	579.33	17,941.02
	Built-up areas	564.52	8169.07	12,811.56	288.63	150.52	308.83	126.18	9607.75
	Forestry land	1438.20	2137.93	304.64	4321.61	22.26	75.06	16.56	3994.64
	Wetland	115.99	501.22	236.51	35.84	1459.46	326.89	30.50	1246.96
	Water areas	152.93	1303.79	404.96	53.25	110.52	5514.69	68.22	2093.67
	Unused land	93.63	752.62	343.28	24.40	40.88	106.09	585.71	1360.91
	Transfer	5739.10	16,646.97	11,685.93	4005.45	907.41	2677.36	941.54	——

	into								
2005–2010	Grassland	9758.47	12.08	45.69	0	9.98	11.25	0.03	79.04
	Cultivated land	14.87	124,856.22	1434.51	27.86	19.70	115.92	6.35	1619.21
	Built-up areas	1.66	196.29	24,233.91	0.39	11.42	39.67	10.57	260.00
	Forestry land	0.97	6.68	17.18	8300.13	1.39	0.72	0	26.94
	Wetland	1.75	14.74	25.51	0.03	2303.65	14.48	0	56.51
	Water areas	1.81	26.29	94.56	0	51.73	8017.04	0.57	174.97
	Unused land	0.88	12.33	116.03	0	0.39	19.11	1378.46	148.74
	Transfer into	21.94	268.40	1733.49	28.27	94.62	201.16	17.53	——
2010–2015	Grassland	9743.36	7.26	23.17	0.25	0	5.36	1.00	37.04
	Cultivated land	22.94	123,912.03	1071.11	23.10	7.57	69.70	7.31	1201.73
	Built-up areas	16.43	196.25	25,751.85	0.70	1.92	2.92	1.05	219.27
	Forestry land	1.16	5.71	21.75	8296.74	0	3.03	0	31.66
	Wetland	0	4.82	9.76	0.25	2452.88	10.96	8.07	33.85
	Water areas	11.09	26.42	50.44	0	24.58	8101.72	2.09	114.62
	Unused land	0	5.21	3.44	1.00	0	3.68	1382.65	13.33
	Transfer into	51.62	245.66	1179.66	25.30	34.08	95.65	19.52	——
2015–2	Grassland	2187.65	4457.35	963.65	1401.82	268.02	444.55	46.80	7582.19

020	Cultivated land	2614.84	96,628.79	17,926.75	2666.21	687.58	3269.99	88.21	27,253.57
	Built-up areas	419.88	13,614.07	9132.38	441.00	248.93	2934.40	49.00	17,707.28
	Forestry land	1275.19	3136.42	776.27	2862.27	57.57	156.03	27.80	5429.28
	Wetland	55.26	839.23	289.21	39.69	743.37	494.26	4.42	1722.07
	Water areas	159.27	2462.45	839.10	87.98	227.69	4367.94	18.81	3795.31
	Unused land	43.48	711.93	174.94	12.41	99.45	326.66	23.47	1368.87
	Transfer into	4567.93	25,221.44	20,969.92	4649.11	1589.24	7625.89	235.05	——

Table S7 Values of all ecosystem services functions in the HAMR (million yuan).

Type	FP	RMP	GR	CR	HR	WT	SC	BM	ALP
2000	2907.09	9054.19	14,100.63	14,354.06	15,687.12	9761.07	15,000.03	15,689.01	7112.21
2005	2896.89	9054.92	14,091.17	14,310.01	15,904.28	9899.61	14,979.03	15,713.93	7158.62
2010	2892.30	9050.72	14,087.20	14,323.38	15,943.39	9933.14	14,970.42	15,714.30	7169.05
2015	2875.51	8999.19	14,013.36	14,278.30	16,002.99	9999.32	14,888.45	15,652.80	7167.84
2020	2860.40	9098.34	14,117.58	14,309.26	16,172.45	10,033.86	14,950.64	15,764.64	7228.95
2000–2005	−10.20	0.73	−9.47	−44.05	217.17	138.55	−21.00	24.92	46.41
2005–2010	−4.59	−4.20	−3.96	13.37	39.10	33.53	−8.61	0.37	10.43
2010–2015	−16.79	−51.53	−73.85	−45.08	59.61	66.18	−81.97	−61.50	−1.21
2015–2020	−15.12	99.16	104.22	30.96	169.45	34.54	62.19	111.84	61.11

* FP-food production; RMP-raw material production; GR-gas regulation; CR-climate regulation; HR-hydrology regulation; WT-waste treatment; SC-soil conservation; BM-biodiversity maintenance; ALP-aesthetic landscape provision.

Table S8 Values of all ecosystem services functions in the HAER (million yuan).

Type	FP	RMP	GR	CR	HR	WT	SC	BM	ALP
2000	2037.82	1561.50	2610.98	3566.80	6266.83	6099.21	3894.61	3784.37	1790.40
2005	1974.85	1537.41	2556.01	3460.97	6359.87	6103.47	3790.12	3735.63	1805.93
2010	1917.74	1514.77	2514.44	3407.54	6332.64	6037.62	3705.27	3679.42	1800.10
2015	1870.44	1484.44	2467.22	3359.42	6276.38	5967.25	3625.53	3616.40	1785.08
2020	1744.88	1445.99	2390.51	3239.08	6127.30	5744.13	3454.53	3493.35	1754.95
2000–2005	−62.97	−24.09	−54.97	−105.83	93.04	4.26	−104.49	−48.74	15.53
2005–2010	−57.11	−22.64	−41.57	−53.44	−27.24	−65.84	−84.85	−56.21	−5.83
2010–2015	−47.31	−30.33	−47.22	−48.12	−56.26	−70.37	−79.74	−63.03	−15.02
2015–2020	−125.56	−38.45	−76.71	−120.34	−149.08	−223.12	−171.00	−123.05	−30.12

Note: FP-food production; RMP-raw material production; GR-gas regulation; CR-climate regulation; HR-hydrology regulation; WT-waste treatment; SC-soil conservation; BM-biodiversity maintenance; ALP-aesthetic landscape provision.

Table S9 Values of all ecosystem services functions in the RAMR (million yuan).

Type	FP	RMP	GR	CR	HR	WT	SC	BM	ALP
2000	6774.26	5877.41	10,235.76	12,451.06	12,862.22	13,606.36	14,896.85	12,876.05	4611.63
2005	6723.39	5856.02	10,191.86	12,387.43	12,894.51	13,586.89	14,812.50	12,828.57	4615.93
2010	6699.25	5847.05	10,177.53	12,388.07	12,870.36	13,556.99	14,778.39	12,804.16	4612.96
2015	6666.16	5826.54	10,144.38	12,362.95	12,919.13	13,576.26	14,719.48	12,773.00	4623.08
2020	6372.44	5756.83	9921.60	11,803.54	12,963.45	13,247.52	14,262.17	12,515.43	4603.63
2000–2005	−50.86	−21.39	−43.90	−63.63	32.30	−19.47	−84.36	−47.48	4.30
2005–2010	−24.14	−8.97	−14.33	0.64	−24.16	−29.91	−34.11	−24.41	−2.97
2010–2015	−33.09	−20.51	−33.15	−25.12	48.77	19.28	−58.90	−31.16	10.13
2015–2020	−293.71	−69.71	−222.79	−559.41	44.31	−328.74	−457.31	−257.57	−19.45

* FP-food production; RMP-raw material production; GR-gas regulation; CR-climate regulation; HR-hydrology regulation;

WT-waste treatment; SC-soil conservation; BM-biodiversity maintenance; ALP-aesthetic landscape provision.

Table S10 Values of all ecosystem services functions in the RAER (million yuan).

Type	FP	RMP	GR	CR	HR	WT	SC	BM	ALP
2000	7944.65	4561.80	8493.04	11,918.93	14,704.64	17,206.82	14,213.92	11,904.94	4348.70
2005	7865.51	4523.50	8379.70	11,685.66	14,951.91	17,291.63	14,028.51	11,821.95	4374.89
2010	7800.27	4498.76	8339.13	11,670.46	14,982.04	17,275.83	13,934.67	11,770.34	4387.12
2015	7748.85	4478.06	8302.83	11,629.73	14,961.96	17,222.35	13,859.33	11,721.15	4383.86
2020	7626.14	4291.32	7858.75	11,088.58	15,136.50	17,296.43	13,271.15	11,287.82	4280.41
2000–2005	−79.14	−38.30	−113.33	−233.27	247.27	84.82	−185.41	−82.99	26.19
2005–2010	−65.24	−24.74	−40.57	−15.20	30.13	−15.80	−93.84	−51.61	12.23
2010–2015	−51.42	−20.70	−36.31	−40.73	−20.08	−53.48	−75.34	−49.19	−3.26
2015–2020	−122.71	−186.75	−444.07	−541.14	174.54	74.08	−588.19	−433.33	−103.45

* FP-food production; RMP-raw material production; GR-gas regulation; CR-climate regulation; HR-hydrology regulation; WT-waste treatment; SC-soil conservation; BM-biodiversity maintenance; ALP-aesthetic landscape provision.
